# Supplementary material for: Capsules with Ileocolonic-Targeted Release of Vitamin B2, B3, and C (ColoVit) Intended for Optimization of Gut Health: Development and Validation of the Production Process
Source: Pharmaceutics. 2023 Apr 28;15(5):1354. doi: 10.3390/pharmaceutics15051354 (PMC10223462; doi:10.3390/pharmaceutics15051354)
Supplement: Supplementary file 1 [file pharmaceutics-15-01354-s001.zip › pharmaceutics-2307056-supplementary.pdf]

Supplementary files

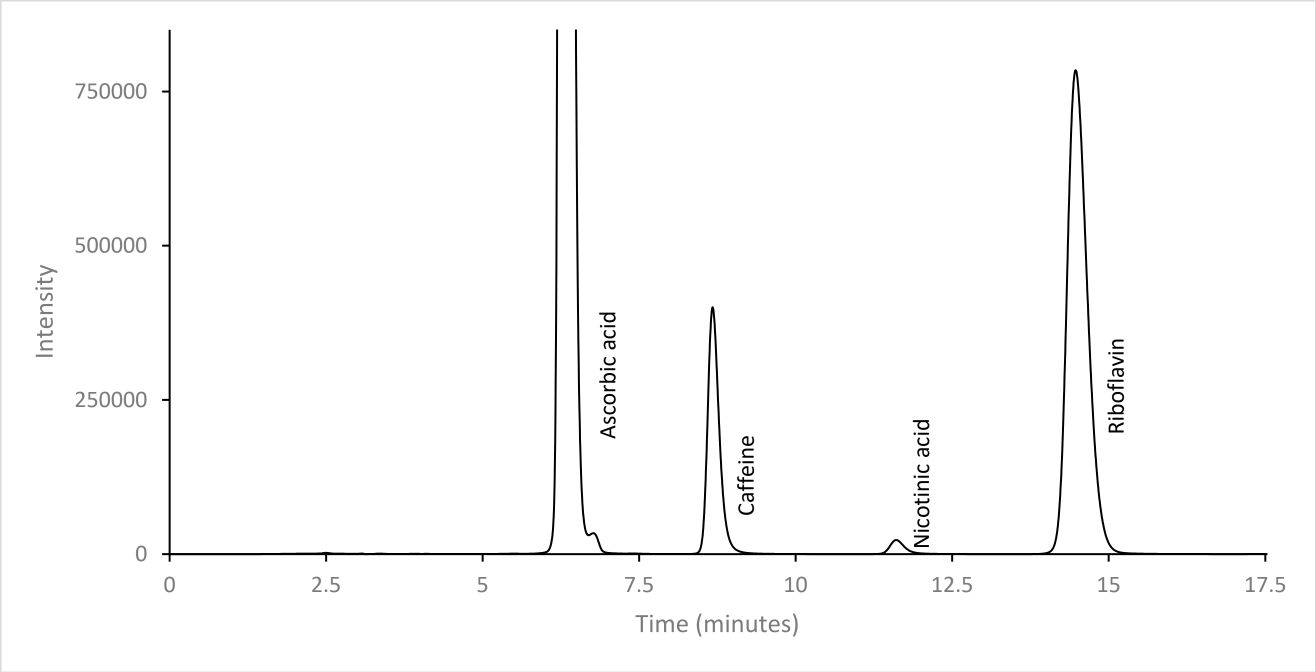

Supplementary Figure S1: Chromatogram of the HPLC analytical assay of caffeine, riboflavin, nicotinic acid, and ascorbic acid
